# The Effect of Ambient Air Pollution during Early Pregnancy on Fetal Ultrasonic Measurements during Mid-Pregnancy

**DOI:** 10.1289/ehp.10720

**Published:** 2007-12-17

**Authors:** Craig A. Hansen, Adrian G. Barnett, Gary Pritchard

**Affiliations:** 1 School of Medicine and; 2 School of Population Health, University of Queensland, Queensland, Australia; 3 PacUser, Paddington, Queensland, Australia

**Keywords:** air pollution, fetal growth, pregnancy, temperature, ultrasound

## Abstract

**Background:**

Over the past decade there has been mounting evidence that ambient air pollution during pregnancy influences fetal growth.

**Objectives:**

This study was designed to examine possible associations between fetal ultrasonic measurements collected from 15,623 scans (13–26 weeks gestation) and ambient air pollution during early pregnancy.

**Methods:**

We calculated mothers’ average monthly exposures over the first 4 months of pregnancy for the following pollutants: particulate matter < 10 μm aerodynamic diameter (PM_10_), ozone, nitrogen dioxide, and sulfur dioxide. We examined associations with fetal femur length (FL), biparietal diameter (BPD), head circumference (HC), and abdominal circumference (AC). Final analyses included scans from only those women within 2 km of an air pollution monitoring site. We controlled for long-term trend, season, temperature, gestation, mother’s age, socioeconomic status, and fetal sex.

**Results:**

A reduction in fetal AC was associated with O_3_ during days 31–60 [−1.42 mm; 95% confidence interval (CI), −2.74 to −0.09], SO_2_ during days 61–90 (−1.67 mm; 95% CI, −2.94 to −0.40), and PM_10_ during days 91–120 (−0.78 mm; 95% CI, −1.49 to −0.08). Other results showed a reduction in BPD (−0.68 mm; 95% CI, −1.09 to −0.27) associated with SO_2_ during days 0–30, a reduction in HC (−1.02 mm; 95% CI, −1.78 to −0.26) associated with PM_10_ during days 91–120, and a reduction in FL associated with PM_10_ during days 0–30 (−0.28 mm; 95% CI, −0.48 to −0.08) and 91–120 (−0.23; 95% CI, −0.42 to −0.04).

**Conclusion:**

We found strong effects of ambient air pollution on ultrasound measures. Future research, including more individually detailed data, is needed to confirm our results.

Over the past decade there has been mounting evidence that ambient air pollution during pregnancy influences fetal growth. Studies conducted throughout North and South America (Chen et al. 2002; Gouveia et al. 2004; Liu et al. 2003; Maisonet et al. 2001; Parker et al. 2005; Ritz and Yu 1999; Rogers and Dunlop 2006; Rogers et al. 2000; Salam et al. 2005), China (Wang et al. 1997), Korea (Ha et al. 2001; Lee et al. 2003), Taiwan (Yang et al. 2003), the Czech Republic (Bobak 2000), and Australia (Mannes et al. 2005) have reported an association between increased concentrations of ambient air pollution during pregnancy and suboptimal fetal growth. However, although it is suggested that ambient air pollution adversely influences fetal growth, there is inconsistency in the strength of the reported effects, and the associated key periods of exposure.

A limitation of environmental studies that use birth weight as a proxy measure of fetal growth is that patterns of fetal growth during pregnancy cannot be assessed. This is particularly important when investigating pollutant exposures during early pregnancy as birth weight is recorded many months after the exposure period. The insult of air pollution may have a transient effect on fetal growth, where growth is hindered at one point in time but then catches up at a later point. For example, maternal smoking during pregnancy can alter the growth rate of individual body segments of the fetus at variable developmental stages, as the fetus experiences selective growth restriction and augmentation (Lampl et al. 2003).

Many studies have reported seasonal patterns in birth weight (Elter et al. 2004; Lawlor et al. 2005; Matsuda et al. 1993; McGrath et al. 2005a, 2005b; Murray et al. 2000; Tustin et al. 2004); however, the authors of these studies postulate that seasonal factors other than air pollution are the result of the seasonal variation in birth weight. For example, in Australia, McGrath and colleagues (2005b) suggested that prenatal vitamin D associated with maternal exposure to sunlight is associated with the seasonal variation in birth weight. The authors of studies in Northern Ireland (Murray et al. 2000), Scotland (Lawlor et al. 2005), and Turkey (Elter et al. 2004) suggest that low birth weight may be attributable to various factors such as changes in maternal diet during pregnancy, increased blood viscosity during pregnancy associated with colder temperatures, or increased levels of growth hormone associated with warmer temperatures. In New Zealand, Tustin and colleagues (2004) found that seasonal variation in birth weight was associated with sunlight during the first trimester and not temperature, and postulated that the positive relationship between sunlight and increased fetal growth is the result of increased levels of an insulin-like growth factor.

The aim of this study was to examine possible associations between fetal ultrasonic measurements and ambient air pollution during early pregnancy. This has two advantages over previous studies: *a*) Fetal growth is assessed during pregnancy as opposed to at birth, and *b*) there is little delay between the exposures and fetal growth measurements, which reduces potential confounding and uses exposures that are concurrent with the observed growth pattern of the fetus.

## Methods

### Fetal ultrasound measurements

We obtained fetal ultrasound measurements from a private ultrasound clinic in Brisbane, Australia. The ultrasound clinic services a large number of pregnant women within the South East Queensland and Northern New South Wales areas. The data were originally collected for a study that constructed population-specific charts of fetal biometry for 11–41 weeks gestation in relation to known gestational age from a large population of normal Australian pregnancies (Schluter et al. 2004). All women attending the clinic for an ultrasound scan between January 1993 and April 2003 were included in the original study, unless they refused to consent to their information being used for research purposes. Referral from obstetricians was based on the established standard of care for “routine ultrasound” between 11 and 41 weeks, so selection into the study was not random. Included in the study were separate scans for 20,555 singleton pregnancies from 17,660 women who had a valid record of their last menstrual period (LMP). Fetal ultrasound biometric measurements were recorded for biparietal diameter (BPD), femur length (FL), abdominal circumference (AC), and head circumference (HC). All readings were recorded in millimeters.

The data used in the current study were the four fetal ultrasound measurements stated above, fetus sex (male, female, unknown), weeks gestation (using the scan date and the mother’s LMP), mother’s age, and mother’s residential postcode. All data were de-identified by assigning a random identification number and the names of the mothers, and other personal details were deleted before data extraction.

There were 23,080 ultrasound scans for which the mother’s postcode at the time of the scan was in Queensland. However, to improve exposure assessment, we further restricted this sample to include only scans from women for whom the centroid of their postcode was within 14 km of an air pollution monitoring site within the Brisbane area. Based on this restriction, there were 19,108 scans eligible for selection into the final analyses. Of these, there were 15,623 (82%) scans during 13–26 weeks gestation (second trimester), and 3,485 (18%) scans at ≥ 27 weeks. To fulfill the main objective of this study, we further restricted our sample to include only the 15,623 scans during weeks 13–26 of gestation, for two reasons: First, there is less time between the exposure periods and when the fetal measurement is recorded, hence leaving less time for other factors to influence fetal growth; and second, this eliminates scans during late pregnancy that may have been performed for problematic pregnancies.

No measure of socioeconomic status (SES) was collected from women at the ultrasound clinic. Their postcode was collected, so we were able to link an area-level index of SES by postcode. This index of SES is the index of relative socioeconomic disadvantage developed by the Australian Bureau of Statistics and is derived from attributes such as low income, low educational attainment, high unemployment, and jobs in relatively unskilled occupations, where a low score indicates socioeconomic disadvantage (Australian Bureau of Statistics 2001a). The index of SES was then grouped by quartiles based on the index of relative socioeconomic disadvantage data for the postcode areas within Queensland (Australian Bureau of Statistics 2001a).

### Air pollution and meteorologic data

For the period 1992 to 2003, air pollution and meteorologic data for Brisbane and surrounding areas were obtained from the Air Services Unit, Queensland Environmental Protection Agency (QEPA; unpublished data). These data were monitored at 18 different sites with the majority located within a 30-km radius of Brisbane city. Hourly readings were obtained for ozone (parts per billion), nitrogen dioxide (parts per billion), sulfur dioxide (parts per billion), and particulate matter with an aerodynamic diameter < 10 μm (PM_10_; reported as micrograms per cubic meter). A daily average was calculated for PM_10_, NO_2_, and SO_2_, whereas an 8-hr average was calculated for O_3_. Not all pollutants were monitored at all 18 sites for the entire study period. Daily average temperature (degrees Celsius) was available from 5 of the QEPA air pollution monitoring sites and from the Queensland Bureau of Meteorology (monitored at Brisbane Airport; unpublished data).

### Exposure assessment

We took the following steps to assign air pollution and temperature exposures to each ultrasound scan. We obtained the digital boundaries of the Queensland postcode areas from the Australian Bureau of Statistics (Australian Bureau of Statistics 2001b) and calculated the distance from the centroid of each postcode to each monitoring site. Given the mother’s residential postcode, we assigned an estimate for each air pollutant and temperature to each day of gestation using the closest monitoring site. If there were missing data from the closest site for a particular day of gestation, then the reading was taken from the next closest site without missing data. If the daily readings were missing across all sites, then the daily exposure estimate was left as missing. This did not occur for O_3_, NO_2_, and temperature, because there were no periods of missing data across all sites.

We then calculated average exposure estimates over the days of gestation for the first 4 individual months (defined as 30-day periods) of gestation. Average distance to the monitoring site was also calculated.

### Data analysis

Most of the ultrasound scans were performed during mid-pregnancy; therefore, the first four 30-day periods after LMP are the exposure periods investigated here. An observation was excluded from the analysis if the exposure was missing for > 5 days (out of 30).

The analysis of the association between air pollutants and ultrasound measurements had four stages. In the first stage we regressed each of the four ultrasound measurements against gestational age, mother’s age, and quartile of SES index. The effect of gestation was nonlinear to capture the average growth curve of a fetus (Schluter et al. 2004). The residuals from this model were then a measure of fetal growth relative to average growth, and were adjusted for mother’s age and SES. By doing this preliminary regression, we ensured that these adjustments were based on all the results (20,080 scans), rather than on the subset of scans with usable exposure data.

For any analysis of air pollution, two of the most important confounders are season and temperature (Dominici et al. 2003). All the air pollutants in this study had a seasonal pattern, and were correlated with temperature (although the strength of these patterns and correlations varied by pollutant). To remove the possibly strong confounder of season, we used a stratified regression technique that is analogous to a case–crossover analysis, but is able to use a normally distributed outcome variable (Barnett et al. 2004). We first partitioned the data into 30-day windows using the date of the scan; this partitioning is the same as that used by the time-stratified case–crossover (Levy et al. 2001). In each window, the individual exposures (pollutants and temperature) were adjusted by subtracting the mean exposure (using all the scans in that window). The residual ultrasound measurements from stage 1 were also adjusted in this way. This technique removes any seasonality and long-term trend from both the exposures and the ultrasound measurements.

To remove the confounding effects of temperature, we next regressed the adjusted air pollutant from stage 2 against the adjusted temperature. The residuals from this model were then the residual effect of air pollution after adjusting for temperature. These residuals and the adjusted temperature were then entered into the final model as possible predictors of fetal growth. The pollutant residuals and adjusted temperature had a correlation of zero, so there was no possibility of confounding. This method assumes that when temperature and pollution are correlated, any association with fetal growth is attributable to temperature. Hence this method gives a conservative estimate of the effect of air pollution.

In the fourth and final stage, the stratified ultrasound residuals were regressed against the adjusted estimates of temperature and pollution and against fetal sex. Sixteen percent of women had more than one ultrasound scan, and to control for the nonindependence of these observations we fitted the regression using a generalized estimating equation with an exchangeable correlation structure (Dobson 2002). The model was fitted using PROC GENMOD in SAS (SAS Institute Inc., Cary, NC, USA). We compared the results of this four-stage model with a simple linear regression of fetal growth on pollutant exposure without adjusting for time, season, or any other covariates.

Changes in the ultrasound measures are shown for an interquartile range (IQR) increase in pollutant; pollutants were entered into the model as continuous covariates. An IQR increase can be thought of as the difference between a moderately good and a moderately bad exposure period. This makes the changes from different pollutants more comparable.

In preliminary analyses, we investigated whether the effect of pollution on growth was nonlinear using penalized splines (Ruppert et al. 2003), but did not find any evidence for nonlinearity. We also investigated the association of SES quartile on each measure of fetal biometry before examining the associations between pollutant exposure and growth. To assess any effect modification by SES, we included an interaction term between SES quartile and exposure.

Those women who lived closer to an air pollution monitor should have had a more accurate assessment of their exposure than did those who lived further away. To assess the effect of this change in measurement accuracy on the association between exposure and fetal growth, we conducted separate analyses using only those scans where the average distance to the monitoring sites was within the following ranges: 2, 4, 6, 8, 10, 12, and 14 km. The number of scans included in each of the final analyses varied, because fewer data were available when we restricted the analyses to only those women living within close proximity of a monitoring site.

To assess the effects of multiple pollutants, we ideally would have matched on secondary pollutant exposures, to completely remove any possible confounding effect of secondary exposures (Barnett et al. 2006). However, matching reduces sample size because those women whose exposures cannot be matched are excluded. Because of our relatively small sample size (for women living within 2 km of a monitor), we instead examined the effect of multiple pollutants by adding an adjusted version of the secondary pollutant to the regression model. The adjusted pollutant was the residual from a linear regression using the predictors of the primary pollutant and temperature. The advantage of this method is that the two-pollutant exposures and temperature are completely uncorrelated. This removes the risk of collinearity in the regression. The disadvantage is that the method assumes that when the two pollutants are correlated, any association with fetal growth is attributable to the primary pollutant (as per stage 3 of the primary analysis). Any association between fetal growth and the secondary pollutant is then that extra association not accounted for by the primary pollutant or temperature.

## Results

There were 15,623 scans during 13–26 weeks of gestation, where the mother resided within 14 km of a monitoring site. These scans were from 14,734 pregnancies where 12,236 (84%) pregnancies had one scan, 1,946 (13%) pregnancies had 2 scans, and 462 (3%) pregnancies had ≥ 3 scans. Tables 1 and 2 show descriptive statistics for the fetal ultrasonic measurements and maternal characteristics. Based on the quartiles of SES, the distribution of women in this study was skewed towards the higher social class. After adjusting for gestational age, and mother’s age, area-level SES had a statistically significant effect only on FL. The lower three classes had an average decrease in FL of between 0.09 and 0.12 mm compared with the highest group.

Descriptive statistics for the air pollution and temperature data during the study period are shown in Table 3. The most complete data for the study period were for NO_2_ and O_3_, which were monitored at most sites, whereas the least complete data were for SO_2_, which was monitored at only 4 of the 18 sites. The table also shows the number of air pollution monitoring sites located within suburbs assigned the index of SES. Many of the monitors are located in areas assigned to the two highest quartiles of SES.

**Figure 1:**
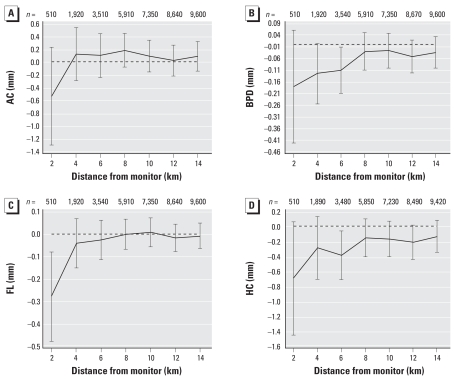
Change in ultrasound measurements (mm) for a 5-μg/m^3^ increase in PM_10_ during days 0–30 of gestation by distance to monitor. (*A*) AC; (*B*) BPD; (*C*) FL; (*D*) HC.

**Figure 2:**
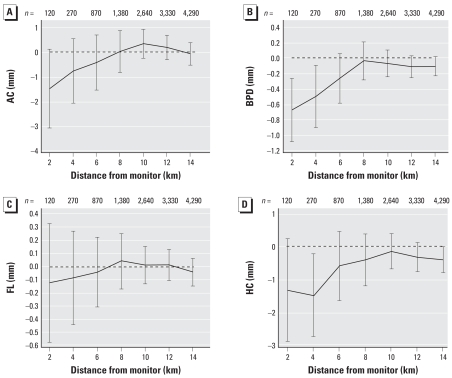
Change in ultrasound measurements (mm) for an 0.8-ppb increase in SO_2_ during days 0–30 of gestation by distance to monitor. (*A*) AC; (*B*) BPD; (*C*) FL; (*D*) HC.

**Figure 3:**
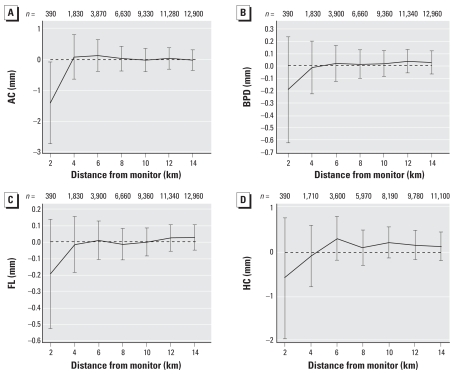
Change in (mm) in ultrasound measurements for an 8-ppb increase in O_3_ during days 31–60 of gestation by distance to monitor. (*A*) AC; (*B*) BPD; (*C*) FL; (*D*) HC.

The change in ultrasound measurements associated with air pollution exposure for a range of distances from monitoring sites is shown for PM_10_ (during days 0–30) (Figure 1); SO_2_ (during days 0–30) (Figure 2); and O_3_ (during days 31–60) (Figure 3). These selected plots show that when increasing air pollution significantly decreased an ultrasound measurement at 2 km, the relationship decreased toward the null as the distance from the monitoring sites increased.

In Figures 1–3, only two associations were statistically significant when using data within 6 km, and no associations were significant when using data beyond this threshold. For most of the results shown, the associations based on data between 8 and 14 km were very similar and were all close to zero. Given this observed pattern in Figures 1–3, the remainder of the results included only scans from women within 2 km of the monitoring sites. The disadvantage of this restriction is a loss of eligible women and hence statistical power, as shown by the wider confidence intervals at shorter distances.

Table 4 shows the change in ultrasound measurements associated with exposure to air pollutants during the first 4 months of pregnancy (adjusted and unadjusted results). The adjusted results showed reductions in AC associated with PM_10_, O_3_, and SO_2_ during different exposure periods. O_3_ during days 31–60 was associated with a 1.42-mm reduction in AC, SO_2_ during days 61–90 was associated with a 1.67-mm reduction in AC, and PM_10_ during days 91–120 was associated with a 0.78-mm reduction. All other ultrasound measurements were negatively associated with only one pollutant each. There was a 1.02-mm reduction in HC associated with PM_10_ during days 91–120, a 0.68-mm reduction in BPD associated with SO_2_ during days 0–30, and a reduction in FL of 0.23–0.28 mm associated with PM_10_ during days 0–30 and 91–120. The unadjusted associations were always smaller than the adjusted associations, and apart from an association between SO_2_ and AC, all unadjusted associations were not significant. There were no statistically significant associations between NO_2_ exposure and any ultrasound measurement.

For each of the significant results in Table 4 we added one of the other pollutants to the model as a secondary pollutant. Except for the effect that O_3_ during days 31–60 had on AC, all of the significant associations for the primary pollutants persisted with very little change in the mean, whereas none of the secondary pollutant effects were statistically significant (Table 5).

To assess any effect modification due to social class, we included an interaction term between exposure and the quartiles of SES (Table 6). There was no strong evidence of effect modification for the effect of PM_10_ during days 91–120 on AC and HC. There was some evidence of effect modification for most of the other associations. In every case the effects of air pollution were stronger in the highest SES quartile.

We also investigated temperature and sunlight exposures using the same methods as used for the air pollutants. For temperature we used scans from women within 2 km of a monitoring site (six sites), and for sunlight we used scans from women within 14 km of the city’s airport where the sunlight readings were collected. We failed to find any significant associations between the temperature, sunlight exposures estimates, and all fetal ultrasonic measurements (results not shown).

## Discussion

In this study we investigated the effect of ambient air pollution during early pregnancy on fetal ultrasonic measures during mid-pregnancy (13–26 weeks gestation). To our knowledge, this is the first study of its kind because it uses ultrasound measurements as direct estimates of growth, rather than using birth weight as an indirect (and delayed) measure of growth.

When analyzing scans from women at different distances to the monitoring sites, we found that if there was a negative relationship between a pollutant and ultrasound measurement, the effect often decreased toward the null when including scans from women who lived further away from the monitoring sites. Because of spatial variation in air pollution, we would expect the measurement error of the pollutant exposure to increase with increasing distance from a monitor. This increase in error causes a decrease in true effects and is known as regression dilution (MacMahon et al. 1990).

This finding highlights the importance of accurate measures of exposure for future studies. Given our results, we recommend using only monitors within a 2-km radius of the subject. We also strongly suspect that our exposure estimates would have been even more accurate (and hence shown larger pollutant effects) if we had each woman’s actual address rather than her postcode. The strong change in the association between air pollution and ultrasound measurements by distance to monitor shown here may also explain the inconsistent results from previous research, which used a range of exposure estimates and distances (e.g., network average vs. close proximity to a monitoring site). Previous air pollution–birth outcome research in Brisbane failed to find reductions in neonate birth weight, head circumference, and length associated with increased ambient air pollution during pregnancy, and this failure may be caused by the maternal exposure estimates being derived from a network average across five monitoring sites (Hansen et al. 2007). When using exposure estimates derived from an average across a network of air pollution monitoring sites, the pollutant effect will most likely be underestimated (Zeger et al. 2000).

Ambient O_3_, SO_2_, and PM_10_ during early pregnancy were associated with reductions in fetal biometry during mid-pregnancy, with the two conspicuous pollutants being PM_10_ and SO_2_. However, in relation to the timing of the exposure and the different fetal body segments, these results were irregular, and until further information is known about the biological mechanisms, it is difficult to associate specific air pollutants with reduced growth of specific fetal body segments. If anything, PM_10_ during days 91–120 showed a consistent pattern across all ultrasound measurements except BPD. This may indicate a sensitive period of gestation in relation to PM_10_ exposure, but it may also be attributed to the strong correlation in ultrasound measurements. Despite the inconsistency in the exposure period, all pollutants except NO_2_ were associated with reductions in abdominal circumference. Based on the premise that the fetus accrues most body fat during the second half of pregnancy, the negative effects on abdominal circumference early in pregnancy suggest that ambient air pollution may interfere with the development of internal organs (e.g., the liver), as abdominal circumference is a proxy measure of the size of these organs (Ville and Nyberg 2003).

Although several biological mechanisms have been suggested, the underlying mechanisms whereby ambient air pollution interferes with fetal growth remain to be determined. It is well recognized that inhalation of air pollutants can cause inflammatory responses and oxidative stress (Donaldson et al. 2001; Kelly 2003; Sorensen et al. 2003), and both of these reactions can interfere with normal intra-uterine growth via vascular dysfunction in the placenta and damaged DNA (Myatt et al. 2000). Also, pro-inflammatory cytokines can limit trophoblast invasion during the early stages of pregnancy, restricting fetal growth (Gitto et al. 2002; Silver et al. 2004). Poor placental vascularity is caused partly by dysregulation of gene expression in key angiogenic factors in early pregnancy (Torry et al. 2004), and if ambient air pollution is associated with poor placental function it may partly be caused by perturbed DNA transcription early in pregnancy (Perera et al. 1999).

In Table 4, the unadjusted associations were always smaller than the adjusted associations. This is most likely explained by seasonal factors. The pollutants that showed an effect had a seasonal pattern that peaked in late spring and summer, whereas the outcomes had weaker seasonal patterns that peaked in spring or winter. This difference in phases would dampen the association between pollutant exposure and fetal biometry. We adjusted for seasonal factors because we believe that the seasonal patterns in pollutants and outcome are exogenous to the year-round association between pollutant and fetal biometry.

We examined the effect of multiple pollutants but were somewhat hampered in this comparison because of the lack of overlap in the pollutant data for all the scans. Hence, when we added a secondary pollutant the sample size decreased (Table 5). Because of this drop in sample size, the statistical power also decreased. Importantly, however, the coefficients for the changes in fetal biometry associated with the primary pollutant remained approximately similar for most of the associations. Hence, the associations found with the single pollutants are relatively robust to other pollutants, and we can be fairly confident that the named pollutants are those attributing to the reductions in fetal biometry. However, air pollution is a complex mixture of compounds, and some of the reported effects may be attributable to other unmeasured pollutants that are correlated with the pollutants analyzed.

Unlike other studies that suggest that seasonal patterns in birth weight are related to ambient temperature (Elter et al. 2004; Lawlor et al. 2005; Murray et al. 2000) and sunlight (Tustin et al. 2004), we failed to find an association between fetal biometry and temperature and sunlight during pregnancy. The lack of effect for sunlight could be attributable to the lack of accurate exposure data, as only the city airport measured hours of sunlight. Also, sunlight exposure depends more on individual behavior than either temperature or air pollution. Whereas a person needs to go outdoors to be exposed to sunlight, this is less true for air pollution, as the air also pervades into homes. This pervasiveness is particularly true in subtropical Brisbane; there is a generally greater need to lose (rather than trap) heat, and many houses are designed to maximize air flow.

This study has a number of important limitations. First, when using residential proximity to a monitoring site as a proxy of exposure, the mother’s postcode at the time of exposure is unknown; it was recorded only at the date of the ultrasound scan. Of the 2,522 pregnancies that had more than one scan, only 176 (7%) had changed postcode from the time of the first scan. This was not a concern because the change of address occurred after the exposure period we investigated. However, for all scans there is the assumption that the postcode at the first scan is the post-code during the first 4 months of pregnancy. This is important because studies investigating maternal residential mobility during pregnancy in relation to birth defects have reported varying rates (12–32%) of women changing address during pregnancy (Canfield et al. 2006; Fell et al. 2004; Khoury et al. 1988; Shaw and Malcoe 1992).

A second limitation is that we had limited data on maternal lifestyle factors such as diet, smoking, and alcohol consumption. When the exposure assessment is based purely on temporal variations in air pollution derived from a network average across a number of sites, these factors do not confound the results because they are constant over time and not associated with the temporal variations in air pollution. However, to improve exposure accuracy we used air pollution data from the closest monitoring site to the women’s post-code area, and this may create residual confounding associated with spatial variation in maternal characteristics that we were unable to control for. We attempted to control for these unknown maternal factors via an index of SES linked to the women’s postcode. Surprisingly, in our preliminary analyses area-level social class had a statistically significant effect only on FL. This may be an indicator that area-level social class is a poor proxy for individual-level social class, and stronger differences would be expected if an individual measure was used. When we included an interaction between exposure and SES quartile, the effect modification of social class on the association between pollutant and fetal biometry was only minor for most associations. Interestingly, the pollutant effects were still significant in the highest social class group for all but two of the results. No pollutant effects were significant in the lower social class group. These effects are somewhat associated with sample size: The highest SES group had the largest sample size, and the lowest SES group the smallest.

A study of the effects of particulate matter on death showed a reduction in risk when adjusting for area-level deprivation (Næss et al. 2007). It is possible that the results shown here would be ameliorated if we had individual SES or a more accurate measure of area-level SES. This would occur if exposure and SES were correlated and lower-SES groups lived in heavily built-up areas with more traffic. This disparity in air pollution exposure has been previously reported among pregnant women (Woodruff et al. 2003). Air pollution monitors are often placed in the areas of highest pollution. These areas might be expected to have the lowest SES, although clearly this is not the case in Brisbane (Table 3). However, there is still the potential of residual confounding as a result of within-area (postcode) variation in both exposure and maternal characteristics that could not be assessed.

Another limitation of this study is that we were unable to obtain subsequent birth outcome data from the state health department for the pregnancies within our study. Therefore, it is difficult to conclude whether these reductions associated with air pollution during early pregnancy persisted until birth and whether there is any clinical relevance to these reductions.

Given the large number of comparisons performed, some of our findings may have occurred by chance (type 1 errors).

Despite these limitations, our study adds to the growing body of literature suggesting that ambient air pollution during pregnancy influences fetal growth. We strongly recommend further exploration of fetal ultrasonic measures in air pollution–birth outcome research to corroborate our findings and examine some of the confounders (e.g., SES, parity) and possible effect modifiers (e.g., air conditioning) that we were unable to examine here. The results shown here suggest that the pollutant monitor needs to be within at least 2 km of the subject, and optimally each woman’s address would be geocoded. We also recommend collecting individual-level data such as distance to major road and time spent outside, as well as accurate data on temperature and sunlight exposure.

Although future work including more individually detailed data is needed to confirm our results, we recommend that pregnant women (where possible) reduce their exposure to air pollution.

## Figures and Tables

**Table 1:** Descriptive statistics for the study population, using scans at 13–26 weeks gestation from women within 14 km of a monitoring site.

Characteristic	Total
No. of scans	15,623
Gestational age [weeks (mean ± SD)]	19 ± 2
Fetal sex (%)	
Female	42
Male	48
Unknown	10
Mother’s age at LMP (years)	
Mean ± SD	32 ± 5
Minimum–maximum	15–45
Quartile of SES (%)	
1 (lowest)	11
2	8
3	21
4 (highest)	60

**Table 2:** Descriptive statistics for the ultrasound measurements and the mean change in measurement by SES, using scans at 13–26 weeks gestation from women within 14 km of a monitoring site.

Characteristic	HC	BPD	AC	FL
No. of scans	12,993	15,623	15,553	15,613
Mean ± SD (mm)	159.4 ± 20.1	43.1 ± 5.5	136.7 ± 20.3	28.4 ± 5.0
Quartile of SES [mean change^a^ (95% CI)]				
1 (lowest)	−0.17 (−0.63 to 0.29)	−0.06 (−0.18 to 0.07)	−0.09 (−0.60 to 0.42)	−0.09 (−0.19 to 0.01)
2	−0.21 (−0.66 to 0.25)	−0.06 (−0.19 to 0.06)	0.07 (−0.44 to 0.58)	−0.14 (−0.24 to −0.04)**
3	−0.09 (−0.41 to 0.24)	0.01 (−0.08 to 0.10)	−0.08 (−0.45 to 0.29)	−0.12 (−0.20 to −0.05)**
4 (highest)	0 (referent)	0 (referent)	0 (referent)	0 (referent)

CI, confidence interval.

a Adjusted for gestational age and mother’s age.

** *p* < 0.01.

**Table 3:** Air pollution and temperature data in Brisbane for the years 1992 to 2003.

Characteristic	PM_10_ (μg/m^3^)	NO_2_ (ppb)	O_3_ (ppb)	SO_2_ (ppb)	Temperature (°C)
No. of monitoring sites	11	17	16	4	6
Days (%) missing data across all sites	604 (14)	0	0	2,657 (61)	0
Mean (IQR)					
All seasons	17.8 (7.8)	9.8 (6.2)	24.8 (9.8)	1.19 (1.00)	20.4 (6.5)
Summer	17.5 (6.8)	6.3 (3.0)	24.2 (11.5)	1.06 (0.84)	25.0 (2.6)
Fall	15.8 (6.9)	10.0 (5.4)	22.3 (8.0)	1.01 (0.88)	20.1 (4.1)
Winter	16.9 (8.5)	13.2 (5.6)	23.0 (6.7)	1.29 (1.06)	15.3 (2.7)
Spring	20.9 (9.4)	9.4 (5.1)	30.0 (8.7)	1.46 (1.03)	20.6 (3.3)
No. of scans (13–26 weeks) within 2 km of a site^a^	1,083	1,117	1,117	665	503
No. of scans (13–26 weeks) within 14 km of a site^a^	13,601	15,623	15,623	11,475	9,265
No. of air pollution monitors within the suburbs assigned to each quartile of SES^b^					
1 (lowest)	3	4	4	1	2
2	1	1	1	0	1
3	4	7	6	2	1
4 (highest)	3	5	5	1	2

Temperature is 24-hr average; O_3_ is 8-hr average.

a Based on the proximity to the site regardless of the availability of data at the time of gestation.

b Based on the postcode of the suburb the air pollution monitor is located within (this is not based on proximity to the centroid of the area).

**Table 4:** Mean change (95% CI) in fetal ultrasonic measurements recorded between 13 and 26 weeks gestation for an IQR increase in maternal exposure to air pollutants during early pregnancy, using ultrasound scans from women within 2 km of a monitoring site.

		PM_10_ (24 hr) (per 5-μg/m^3^ increase)		NO_2_ (24 hr) (per 5-ppb increase)		O_3_ (8 hr) (per 8-ppb increase)		SO_2_ (24 hr) (per 0.8-ppb increase)	
Measure (mm)	Exposure period (days)	Unadjusted^a^	Adjusted^b^	Unadjusted^a^	Adjusted^b^	Unadjusted^a^	Adjusted^b^	Unadjusted^a^	Adjusted^b^
HC	0–30	−0.15	−0.70	0.47	0.27	−0.34	−0.32	−0.91	−1.33
		(−0.67 to 0.37)	(−1.45 to 0.06)	(−0.27 to 1.21)	(−0.94 to 1.47)	(−1.27 to 0.59)	(−1.56 to 0.91)	(−2.27 to 0.46)	(−2.90 to 0.24)
	31–60	0.30	−0.12	0.36	−0.08	−0.53	−0.58	−0.91	−0.42
		(−0.26 to 0.85)	(−0.79 to 0.54)	(−0.45 to 1.18)	(−1.27 to 1.10)	(−1.69 to 0.64)	(−1.97 to 0.80)	(−2.26 to 0.43)	(−1.77 to 0.94)
	61–90	0.09	−0.14	0.51	−0.30	−0.32	0.26	−1.08	−0.80
		(−0.41 to 0.60)	(−0.91 to 0.63)	(−0.34 to 1.37)	(−1.59 to 1.00)	(−1.46 to 0.81)	(−1.07 to 1.59)	(−2.38 to 0.22)	(−2.36 to 0.76)
	91–120	−0.25	−1.02**	0.33	−0.15	0.13	0.11	0.00	0.03
		(−0.86 to 0.37)	(−1.78 to −0.26)	(−0.37 to 1.03)	(−1.15 to 0.84)	(−1.05 to 1.31)	(−0.98 to 1.21)	(−1.16 to 1.17)	(−1.10 to 1.16)
BPD	0–30	−0.15	−0.18	0.14	0.07	−0.21	−0.15	−0.38	−0.68**
		(−0.31 to 0.00)	(−0.43 to 0.06)	(−0.09 to 0.37)	(−0.31 to 0.44)	(−0.50 to 0.07)	(−0.49 to 0.19)	(−0.79 to 0.03)	(−1.09 to −0.27)
	31–60	−0.04	−0.14	0.14	−0.10	−0.30	−0.20	−0.26	−0.26
		(−0.20 to 0.12)	(−0.33 to 0.05)	(−0.10 to 0.38)	(−0.44 to 0.25)	(−0.65 to 0.04)	(−0.63 to 0.23)	(−0.66 to 0.13)	(−0.66 to 0.14)
	61–90	−0.04	−0.08	0.20	−0.06	−0.05	0.21	−0.05	−0.18
		(−0.19 to 0.11)	(−0.27 to 0.11)	(−0.05 to 0.45)	(−0.41 to 0.29)	(−0.39 to 0.29)	(−0.24 to 0.67)	(−0.46 to 0.35)	(−0.61 to 0.26)
	91–120	−0.03	−0.16	0.13	−0.08	0.18	0.22	0.11	−0.03
		(−0.22 to 0.15)	(−0.38 to 0.06)	(−0.10 to 0.35)	(−0.37 to 0.21)	(−0.17 to 0.52)	(−0.14 to 0.58)	(−0.28 to 0.50)	(−0.38 to 0.31)
AC	0–30	−0.01	−0.54	0.08	0.24	−0.29	−0.63	−1.23	−1.49
		(−0.53 to 0.51)	(−1.30 to 0.23)	(−0.66 to 0.82)	(−0.99 to 1.47)	(−1.20 to 0.63)	(−1.66 to 0.40)	(−2.60 to 0.15)	(−3.09 to 0.11)
	31–60	0.33	0.06	0.13	0.49	−0.80	−1.42*	−1.01	−0.47
		(−0.23 to 0.88)	(−0.61 to 0.72)	(−0.69 to 0.95)	(−0.70 to 1.67)	(−1.99 to 0.39)	(−2.74 to −0.09)	(−2.43 to 0.41)	(−1.93 to 0.98)
	61–90	0.04	−0.19	0.30	0.10	−0.70	0.39	−1.36*	−1.67**
		(−0.47 to 0.56)	(−0.86 to 0.47)	(−0.55 to 1.14)	(−1.06 to 1.26)	(−1.85 to 0.44)	(−1.24 to 2.01)	(−2.63 to −0.09)	(−2.94 to −0.40)
	91–120	−0.26	−0.78*	0.08	0.15	−0.19	0.33	−0.11	−0.18
		(−0.87 to 0.36)	(−1.49 to −0.08)	(−0.64 to 0.81)	(−0.90 to 1.20)	(−1.39 to 1.00)	(−0.97 to 1.62)	(−1.42 to 1.20)	(−1.67 to 1.31)
FL	0–30	−0.12	−0.28**	0.04	0.03	0.00	−0.03	−0.15	−0.12
		(−0.26 to 0.02)	(−0.48 to −0.08)	(−0.15 to 0.23)	(−0.25 to 0.31)	(−0.24 to 0.25)	(−0.32 to 0.27)	(−0.54 to 0.24)	(−0.58 to 0.33)
	31–60	0.04	−0.04	0.01	−0.09	−0.11	−0.20	−0.21	0.05
		(−0.10 to 0.18)	(−0.21 to 0.12)	(−0.20 to 0.22)	(−0.39 to 0.22)	(−0.41 to 0.19)	(−0.53 to 0.14)	(−0.56 to 0.14)	(−0.30 to 0.40)
	61–90	−0.05	−0.16	0.09	0.01	−0.06	0.08	−0.33	−0.25
		(−0.19 to 0.08)	(−0.35 to 0.03)	(−0.13 to 0.30)	(−0.26 to 0.28)	(−0.35 to 0.22)	(−0.35 to 0.51)	(−0.68 to 0.01)	(−0.62 to 0.13)
	91–120	−0.13	−0.23*	−0.02	−0.13	−0.09	0.02	−0.23	−0.22
		(−0.29 to 0.03)	(−0.42 to −0.04)	(−0.21 to 0.16)	(−0.40 to 0.13)	(−0.38 to 0.20)	(−0.30 to 0.34)	(−0.55 to 0.10)	(−0.61 to 0.17)

a Simple linear regression.

b Adjusted for fetal sex, gestational age, mother’s age, mother’s SES, concurrent temperature exposures, and seasonality and long-term trend.

* *p* < 0.05.

** *p* < 0.01.

**Table 5:** Two-pollutant models: statistically significant adjusted pollutant effects and effects after adding a second pollutant.

Pollutant	FL, PM_10_ (0–30 days)	FL, PM_10_ (91–120 days)	AC, PM_10_ (91–120 days)	HC, PM_10_ (91–120 days)	AC, O_3_ (31–60 days)	BPD, SO_2_ (0–30 days)	AC, SO_2_ (61–90 days)
Primary pollutant^a^	*n* = 510	*n* = 510	*n* = 510	*n* = 510	*n* = 390	*n* = 120	*n* = 150
(as per Table 4)	−0.28 (−0.48 to −0.08)**	−0.23 (−0.42 to −0.04)*	−0.78 (−1.49 to −0.08)*	−1.02 (−1.78 to −0.26)**	−1.42 (−2.74 to −0.09)*	−0.68 (−1.09 to −0.27)**	−1.67 (−2.94 to −0.40)*
Two pollutants^a^							
PM_10_	—	—	—	—	*n* = 387	*n* = 120	*n* = 150
Primary					−1.19 (−2.54 to 0.16)	−0.68 (−1.09 to −0.27)**	−1.67 (−2.94 to −0.40)*
Secondary					0.50 (−0.55 to 1.54)	−0.02 (−0.48 to 0.45)	0.20 (−1.13 to 1.53)
O_3_	*n* = 501	*n* = 500	*n* = 500	*n* = 500	—	*n* = 120	*n* = 143
Primary	−0.29 (−0.50 to −0.09)**	−0.23 (−0.43 to −0.04)*	−0.73 (−1.44 to −0.03)*	−1.05 (−1.83 to −0.28)**		−0.67 (−1.09 to −0.25)**	−1.82 (−3.21 to −0.42)*
Secondary	0.01 (−0.35 to 0.36)	−0.07 (−0.37 to 0.23)	−0.28 (−1.48 to 0.92)	0.54 (−0.56 to 1.65)		−0.52 (−1.53 to 0.50)	1.26 (−1.54 to 4.07)
SO_2_	*n* = 464	*n* = 459	*n* = 459	*n* = 459	*n* = 298	—	—
Primary	−0.24 (−0.44 to −0.04)*	−0.21 (−0.40 to −0.01)*	−0.75 (−1.48 to −0.03)*	−0.98 (−1.74 to −0.21)*	−0.81 (−2.29 to 0.66)		
Secondary	0.04 (−0.23 to 0.32)	−0.10 (−0.39 to 0.18)	0.28 (−0.82 to 1.37)	−0.30 (−1.34 to 0.74)	−0.61 (−1.96 to 0.75)		

Cells show sample size (first row), primary pollutant effect (second row), and secondary pollutant effect (third row). Pollutant effects are a mean change in outcome for an IQR increase in pollutant (95% CI).

a Adjusted for fetal sex, gestational age, mother’s age, mother’s SES, concurrent temperature exposures, and seasonality and long-term trend.

* *p* < 0.05.

** *p* < 0.01.

**Table 6:** Statistically significant adjusted pollutant effects and effect-modified estimates by SES quartile.

Pollutant	FL, PM_10_ (0–30 days)	FL, PM_10_ (91–120 days)	AC, PM_10_ (91–120 days)	HC, PM_10_ (91–120 days)	AC, O_3_ (31–60 days)	BPD, SO_2_ (0–30 days)	AC, SO_2_ (61–90 days)
Adjusted^a^ (as per table 4)	−0.28 (−0.48 to −0.08)**	−0.23 (−0.42 to −0.04)*	−0.78 (−1.49 to −0.08)*	−1.02 (−1.78 to −0.26)**	−1.42 (−2.74 to −0.09)*	−0.68 (−1.09 to −0.27)**	−1.67 (−2.94 to −0.40)*
Pollutant effects modified by SES quartile							
1 (lowest)	−0.19 (0.51 to 0.12)	−0.17 (−0.41 to 0.08)	−0.10 (−0.97 to 0.76)	−0.60 (−1.62 to 0.42)	−2.18 (−5.39 to 1.02)	NA	NA
2	−0.29 (−0.55 to −0.03)*	−0.24 (−0.54 to 0.07)	−0.16 (−1.27 to 0.95)	−0.58 (−1.74 to 0.59)	−1.33 (−3.17 to 0.50)	NA	NA
3	−0.21 (−0.47 to 0.06)	−0.20 (−0.46 to 0.05)	−0.14 (−1.04 to 0.76)	−0.40 (−1.47 to 0.67)	−0.94 (−2.33 to 0.45)	−0.65 (−1.06 to −0.23)**	−1.33 (−2.65 to −0.02)*
4 (highest)	−0.26 (0.10 to −0.05)*	−0.23 (−0.44 to −0.02)*	−0.62 (−1.36 to 0.12)	−0.81 (−1.68 to 0.06)	−1.50 (−2.78 to −0.22)*	−0.79 (−1.24 to −0.34)**	−2.29 (−3.65 to −0.94)**

NA, not available. Cells show mean change (mm) in outcome for an IQR increase in pollutant (95% CI).

a Adjusted for fetal sex, gestational age, mother’s age, mother’s SES, concurrent temperature exposures, and seasonality and long-term trend.

* *p* < 0.05.

** *p* < 0.01.
